# Atlas of the I_2_ Spectrum from 19 000 to 18 000 cm^−1^ *

**DOI:** 10.6028/jres.081A.006

**Published:** 1977-02-01

**Authors:** J. D. Simmons, J. T. Hougen

**Affiliations:** Institute for Basic Standards, National Bureau of Standards, Washington, D. C. 20234

**Keywords:** High-resolution spectrum, iodine spectrum, line identification atlas, rovibronic assignments, spectral analysis, visible absorption spectroscopy

## Abstract

A line identification band atlas is presented for a 1000 cm^−1^ segment, from 19 000 to 18 000 cm^−1^, of the molecular iodine absorption spectrum. Each page of the atlas covers a 20 cm^−1^ region of the spectrum and contains a CALCOMP produced photodensitometer trace of the spectrum together with accompanying tabular identification data. The tabular data includes: line identification numbers, observed wavenumbers, calculated wavenumbers, and rotational and vibrational assignments.

## 1. Introduction

The present article represents the first part of a projected band atlas of the $B^{3}\Pi_{0}{+}_{u}-X^{1}\Sigma_{g}^{+}$ visible absorption spectrum of the iodine molecule. The region from 19 000 to 18 000 cm^−1^ (from 5261.7 to 5554.0 Å) was chosen for initial study because it exhibits neither the complications of many close-lying upper state vibrational levels found at higher wavenumbers nor the complications of strong hot bands found at lower wavenumbers.

The visible spectrum of I_2_ has been extensively studied in the past, of course, and it is not our purpose here to trace the numerous developments in the understanding of that spectrum. Suffice it to say that for the present atlas we have relied heavily on the paper by Wei and Tellinghuisen [1].^1^ Measurements here for *J* < 100 agree with the spectrum calculated from the constants of [1] to within ±0.03 cm^−1^ in most cases and ±0.01 cm^−1^ in many cases. The vibrational numbering adopted by Wei and Tellinghuisen and used also in the present atlas is that determined by Steinfeld, Zare, Jones, Lesk, and Klemperer [2] and confirmed by Brown and James [3].

**Figure 1:**
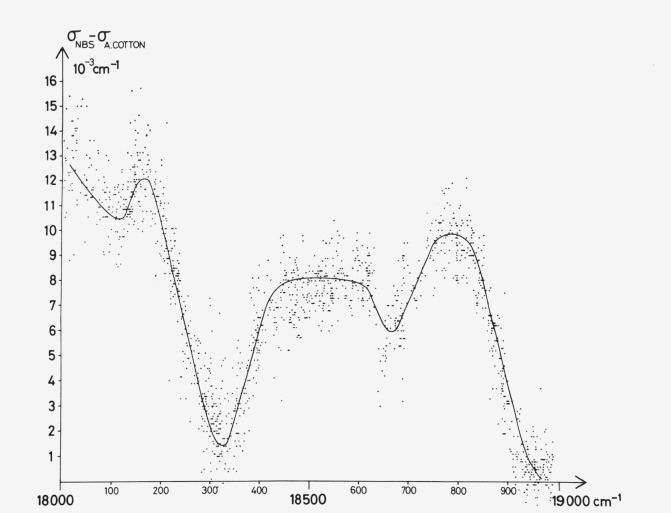
A correction curve for the wavenumbers in this atlas kindly supplied by Gerstenkorn and Luc [5], who plot differences between our grating measurements of the *I_2_* absorption spectrum (*σ_NBS_*) and their interferometric measurements (*σ_A.COTTON_*).

We have very recently learned, through a preprint from Gerstenkorn, Luc, and Perrin [4] on the 5350 Å band of iodine and through subsequent correspondence [5], that a study similar to ours is being carried out at the Laboratoire Aimé Cotton in France. The French investigators have measured the iodine visible spectrum interferometrically, obtaining significantly better absolute measurement accuracy, though no appreciable difference in spectral resolution, since the latter is limited in both studies by the molecular line widths. We have received permission [5] to reproduce here (in fig. 1) their display of differences between our measurements and theirs.

**Figure 2:**
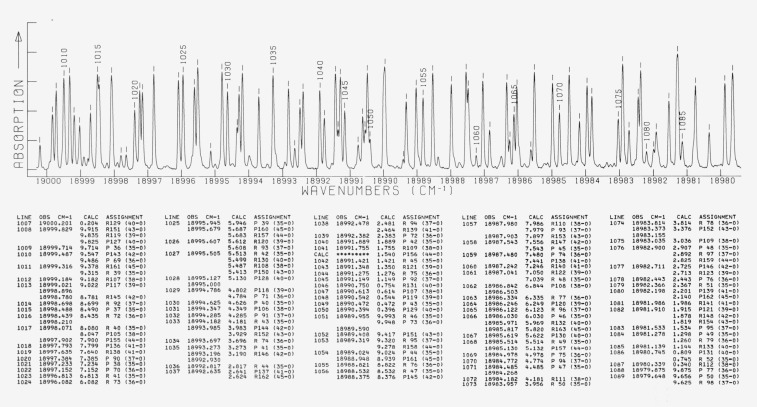

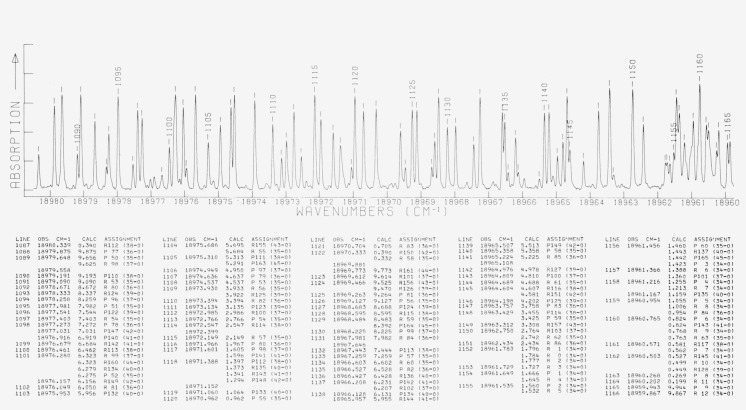

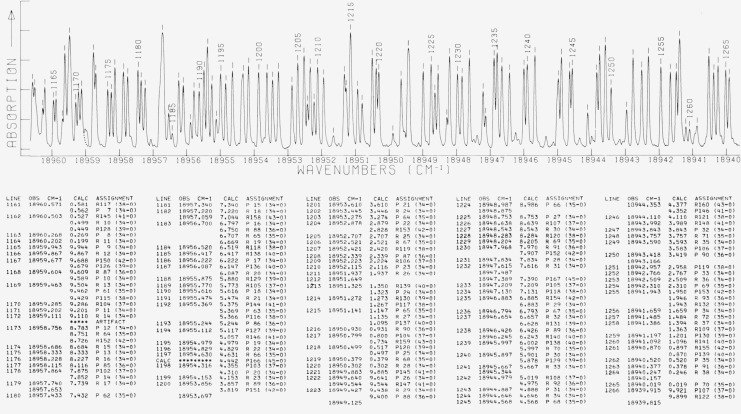

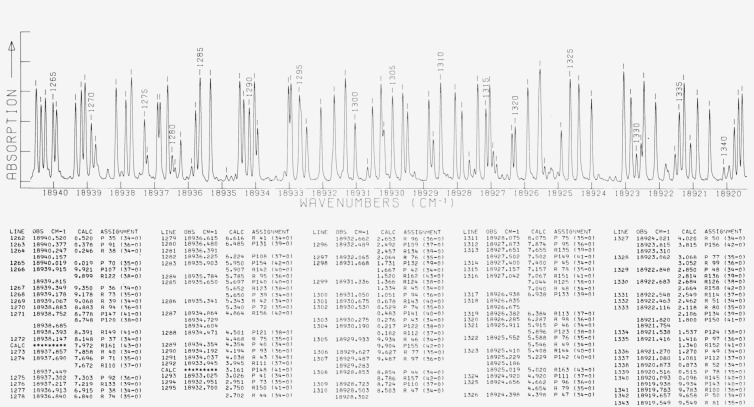

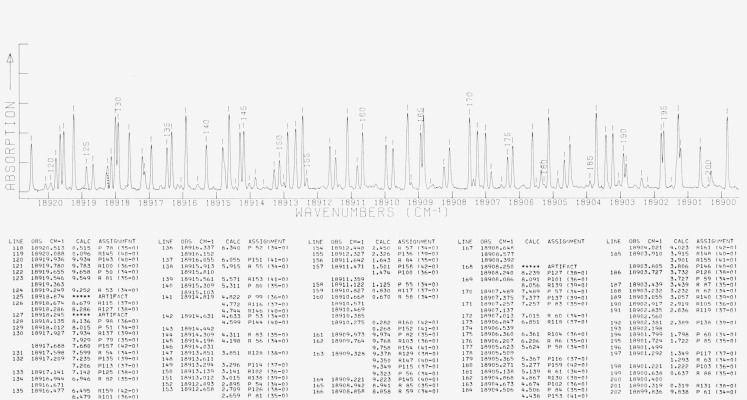

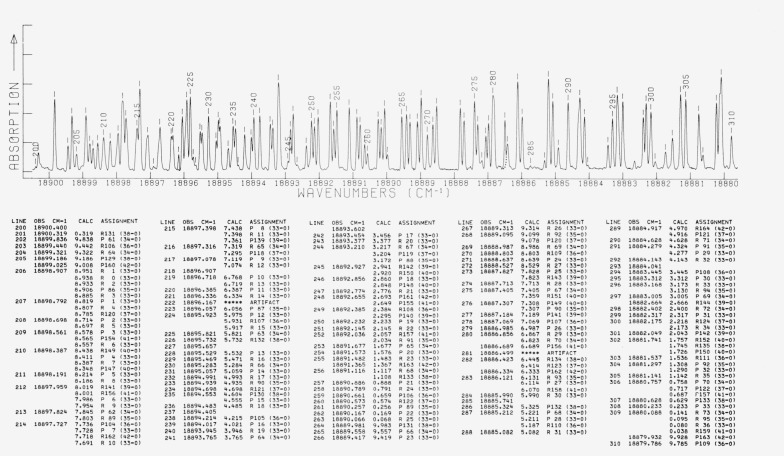

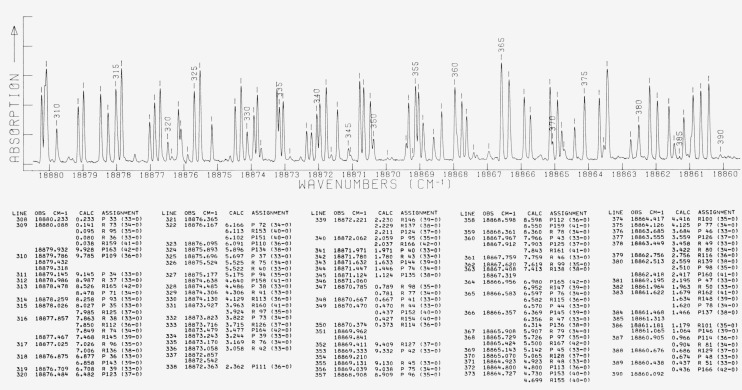

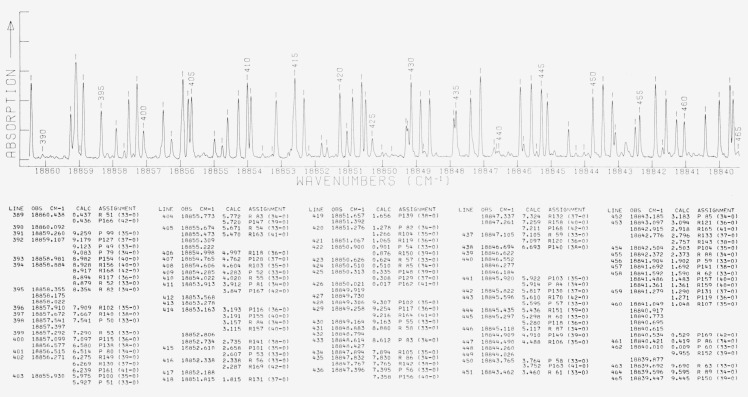

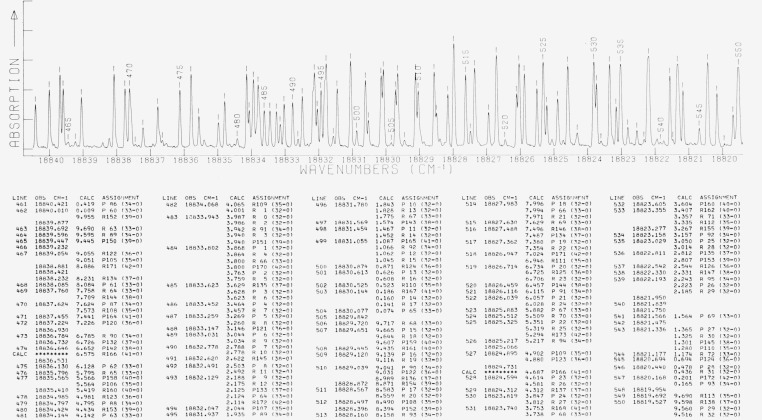

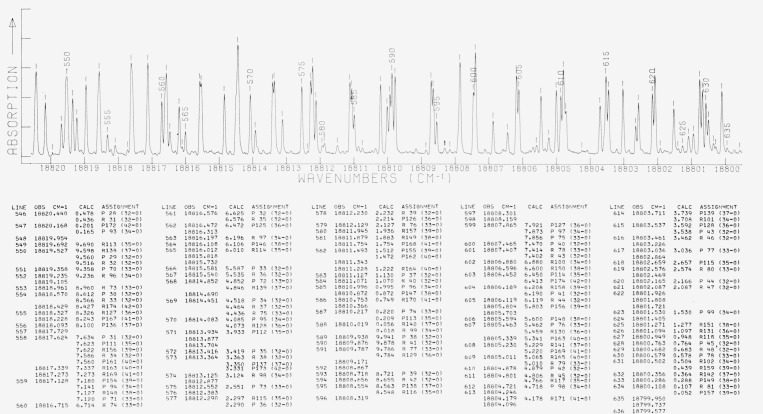

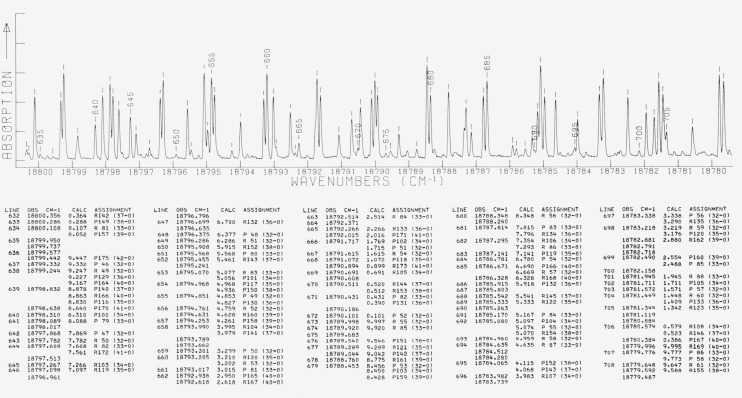

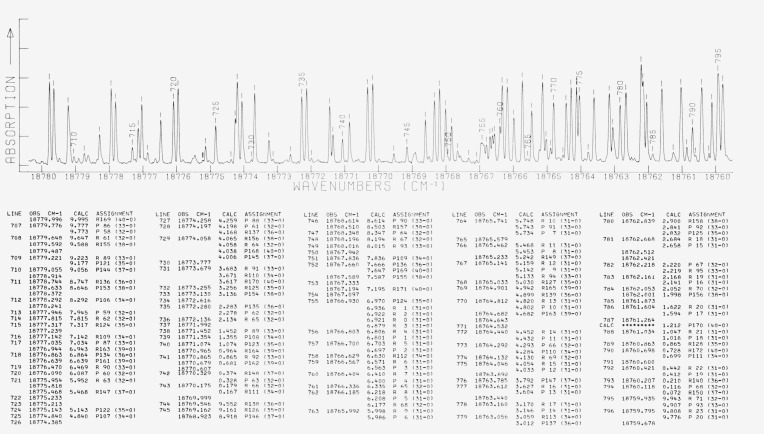

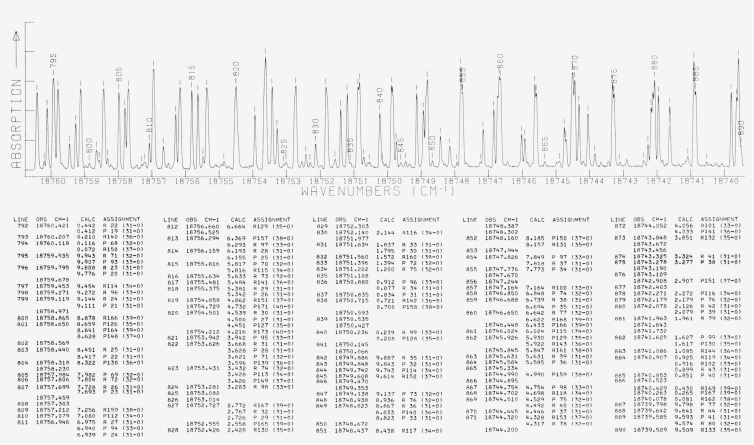

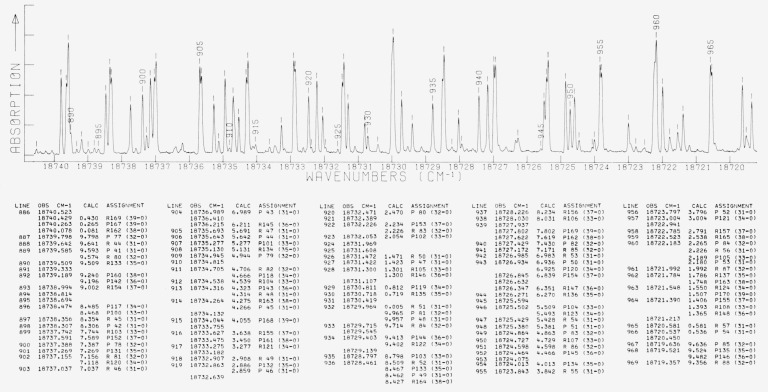

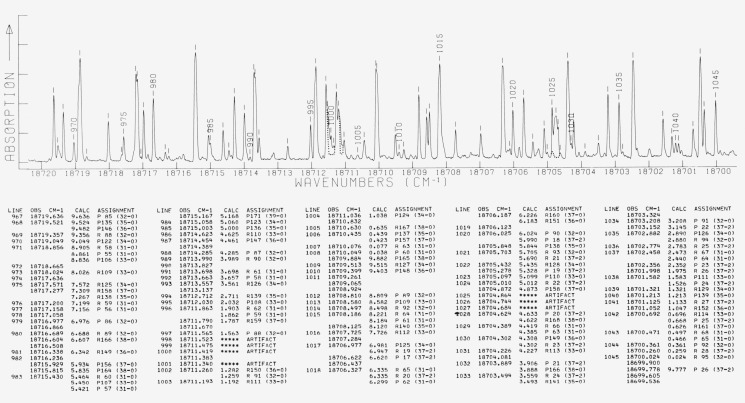

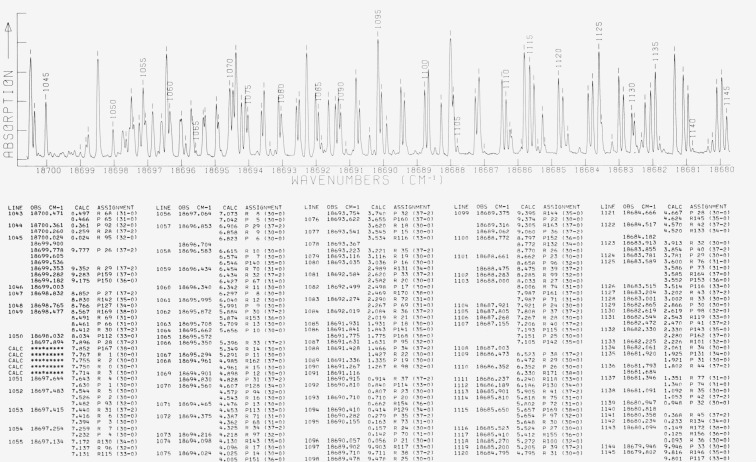

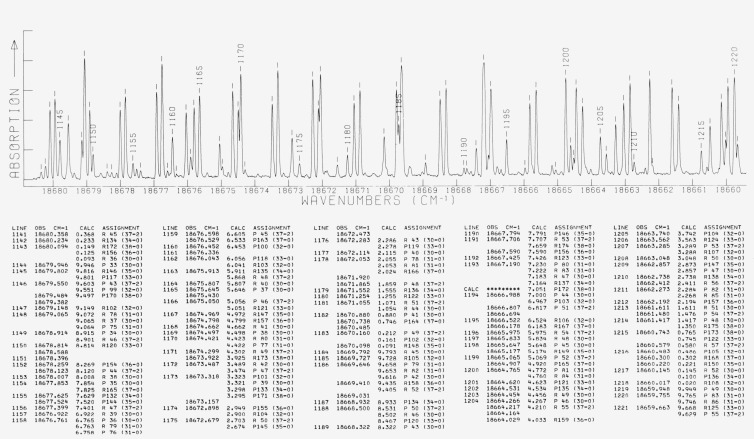

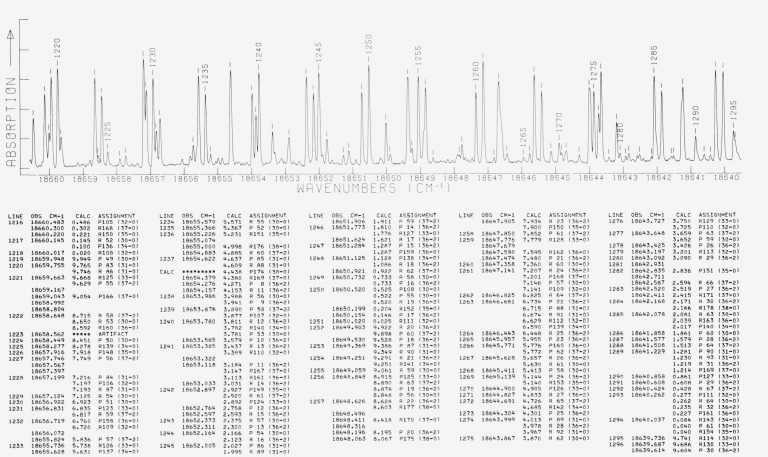

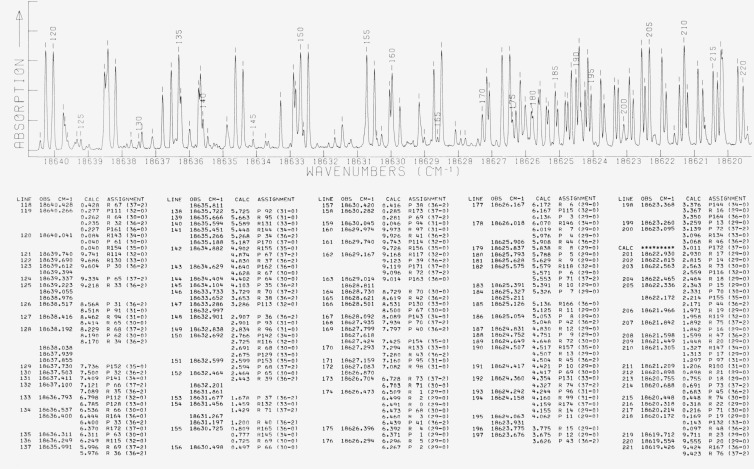

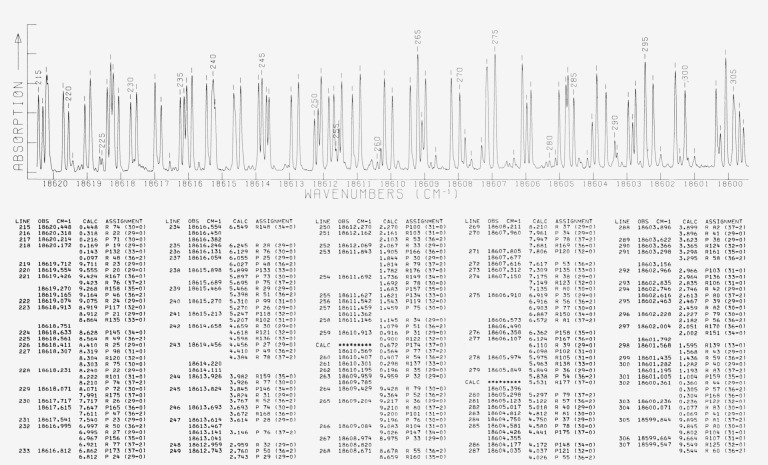

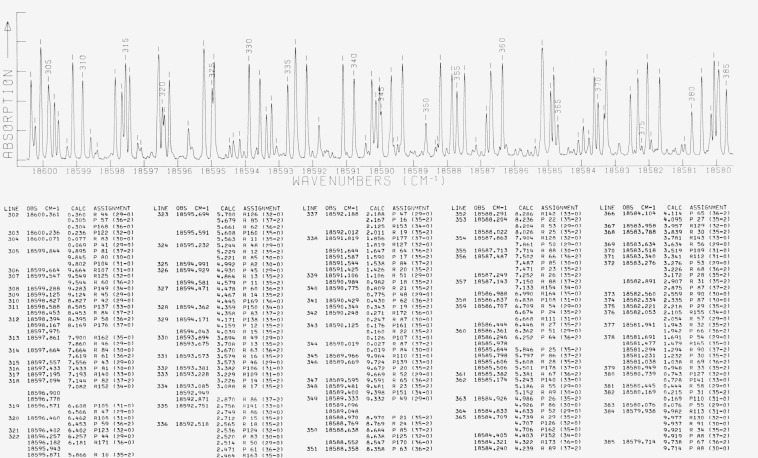

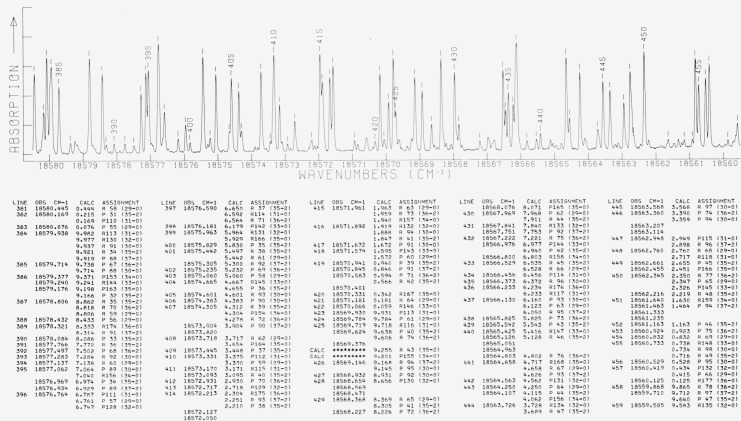

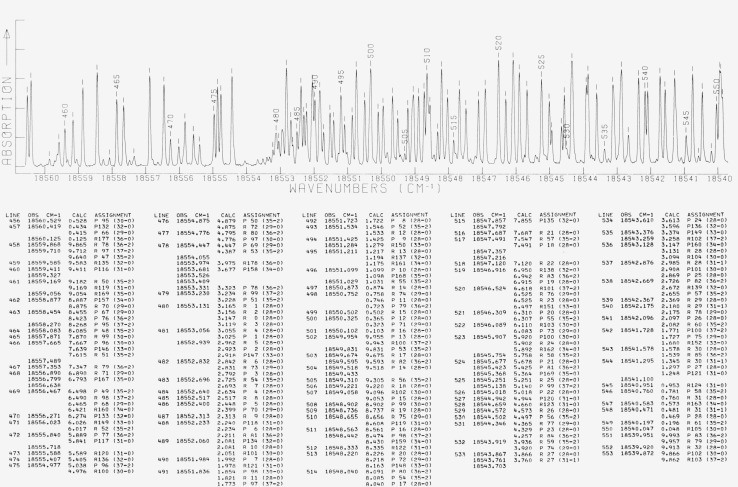

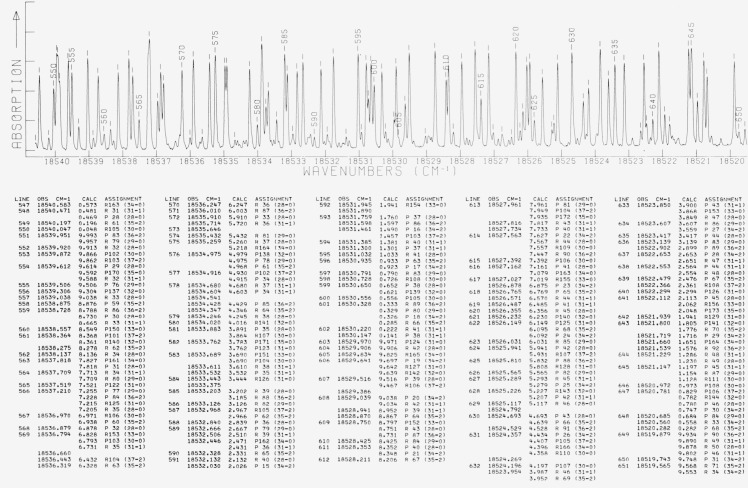

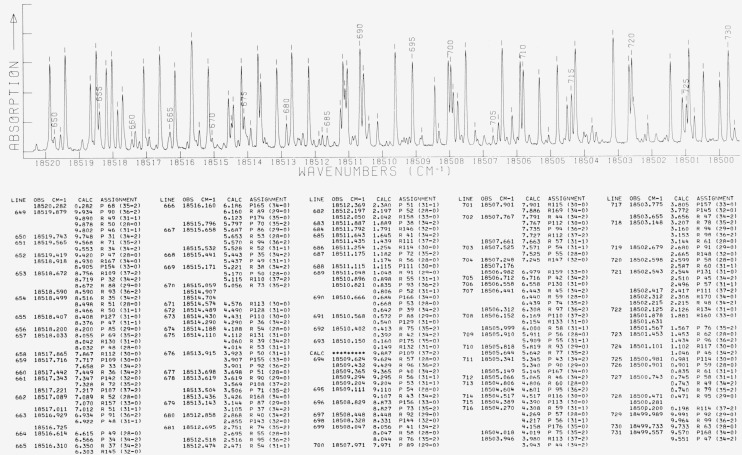

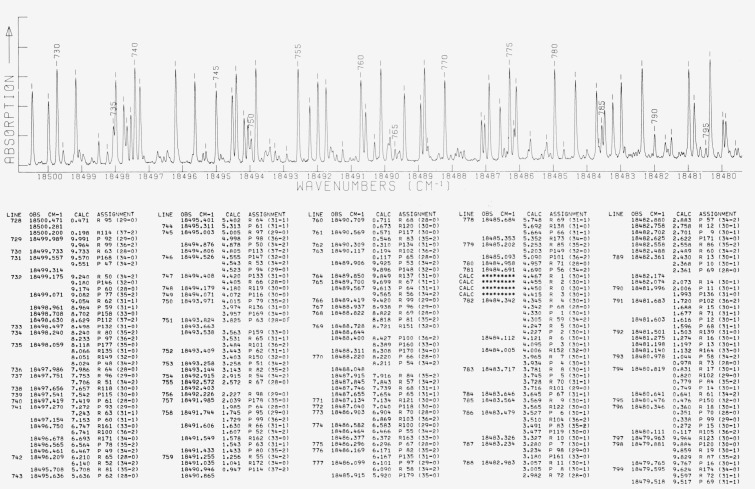

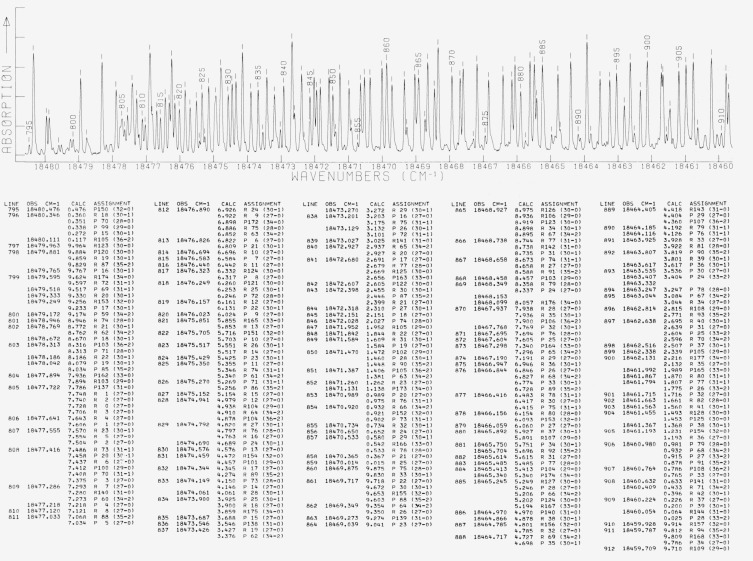

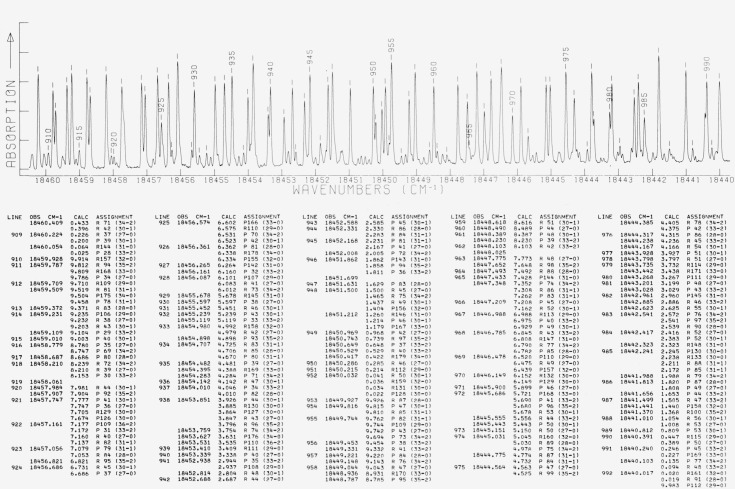

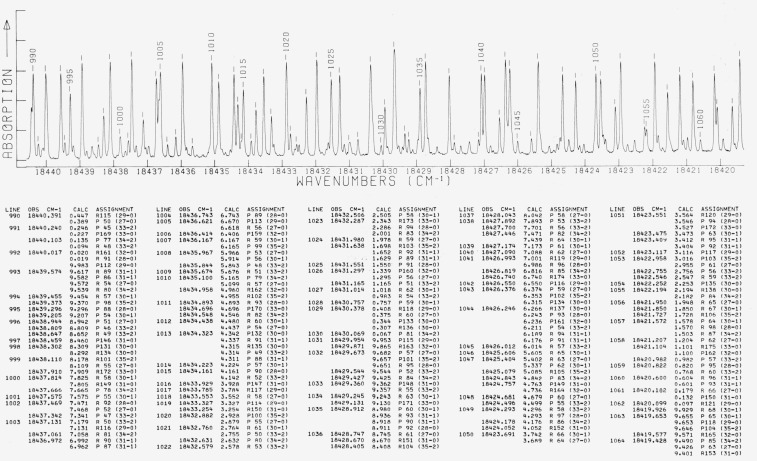

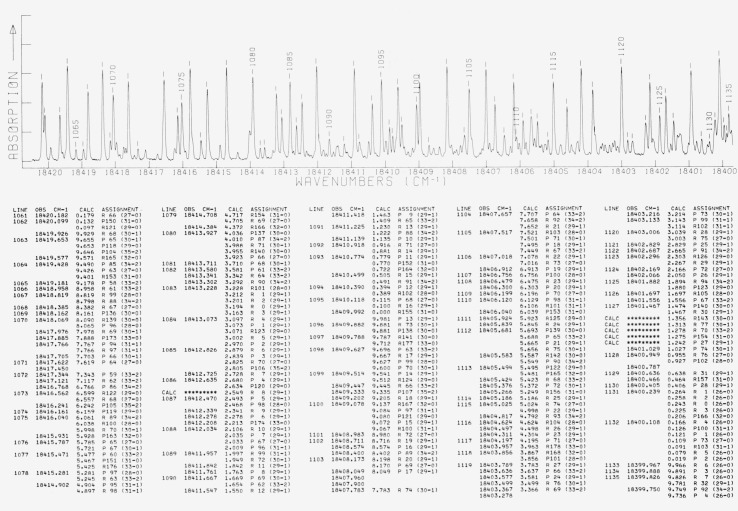

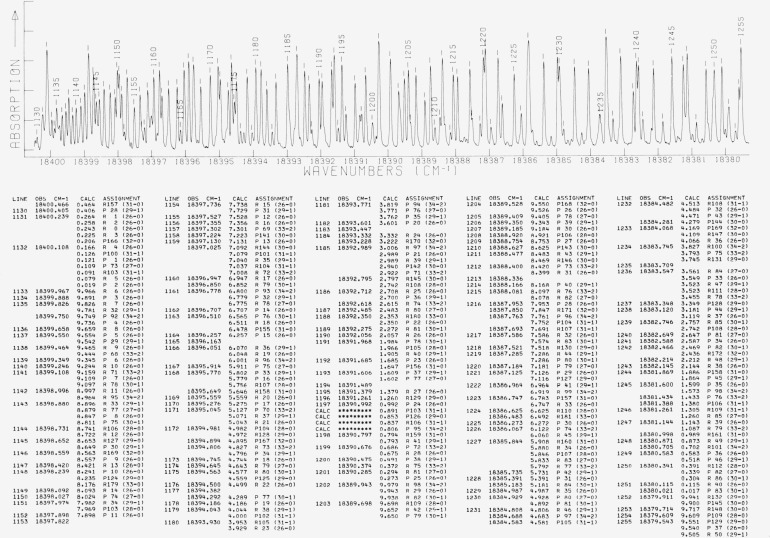

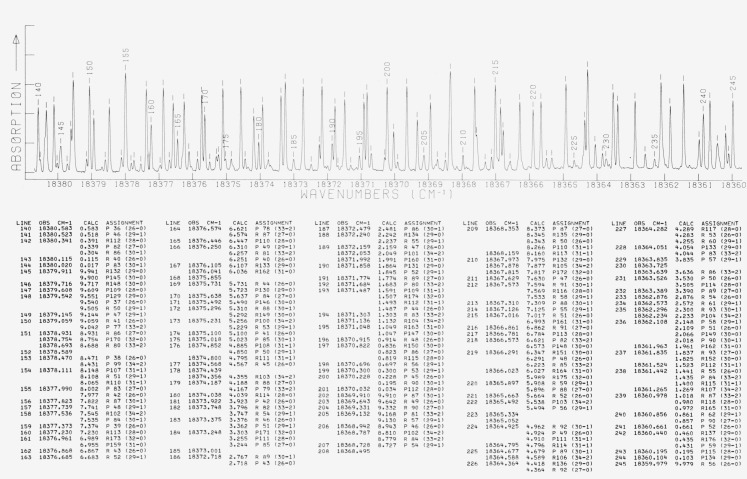

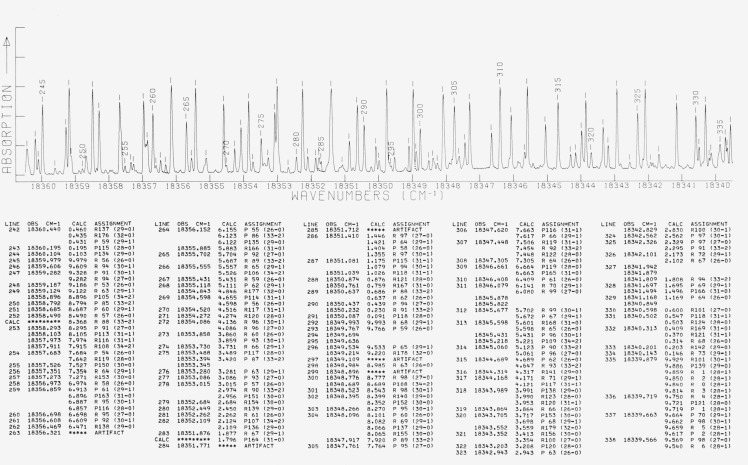

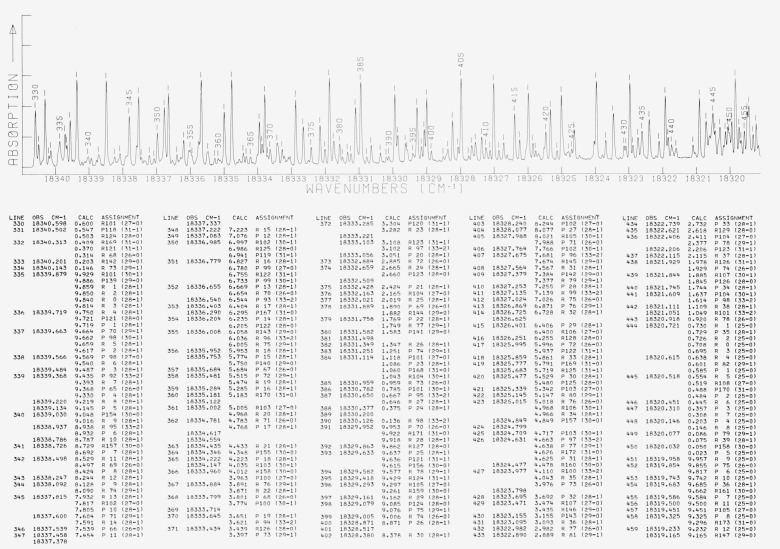

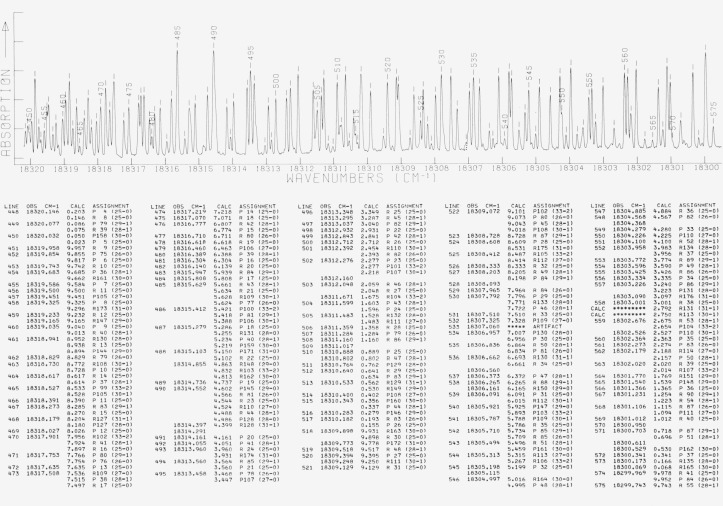

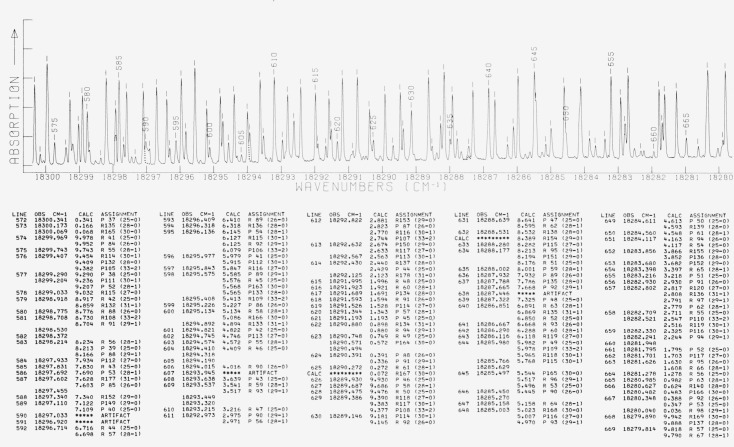

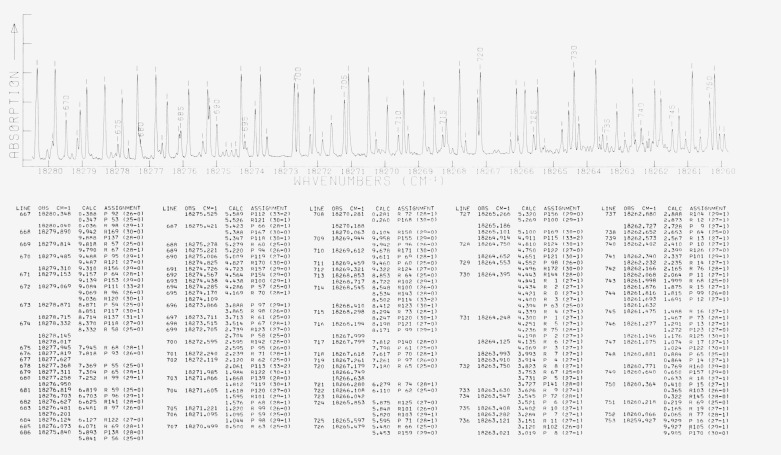

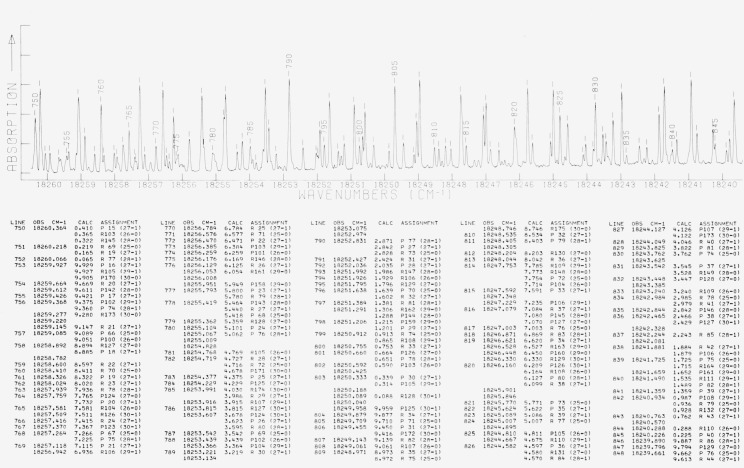

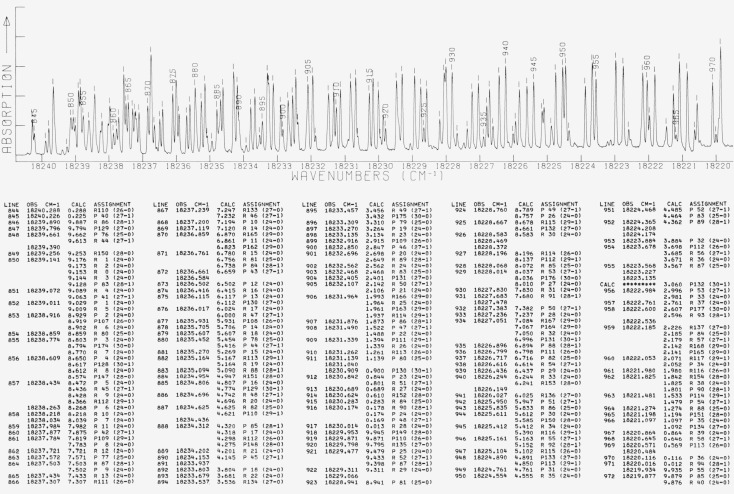

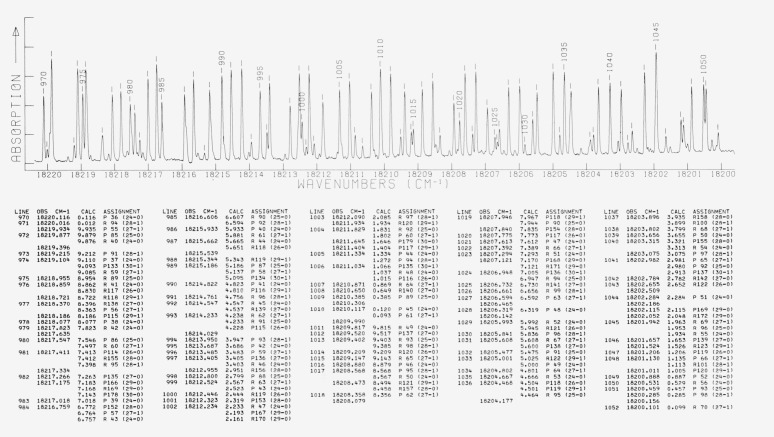

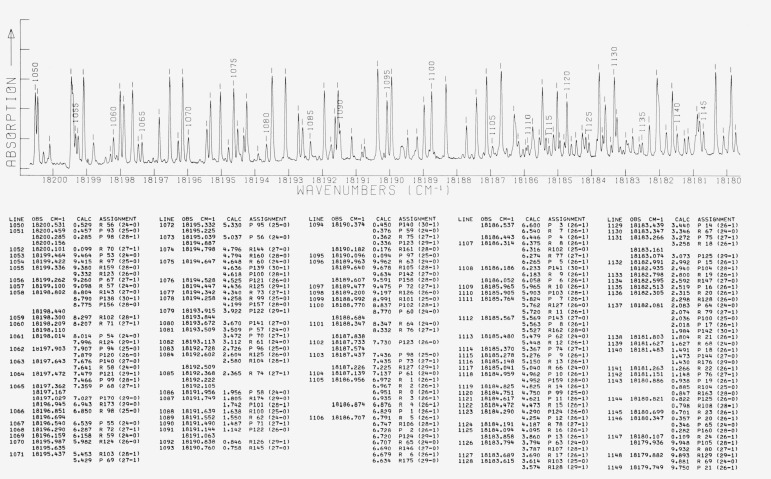

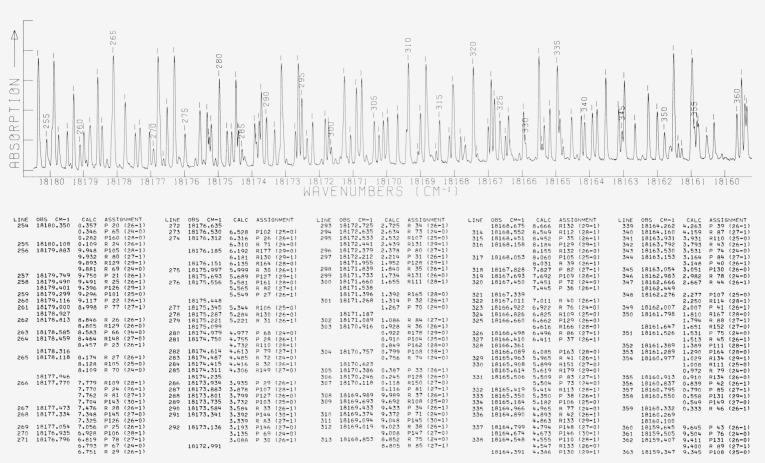

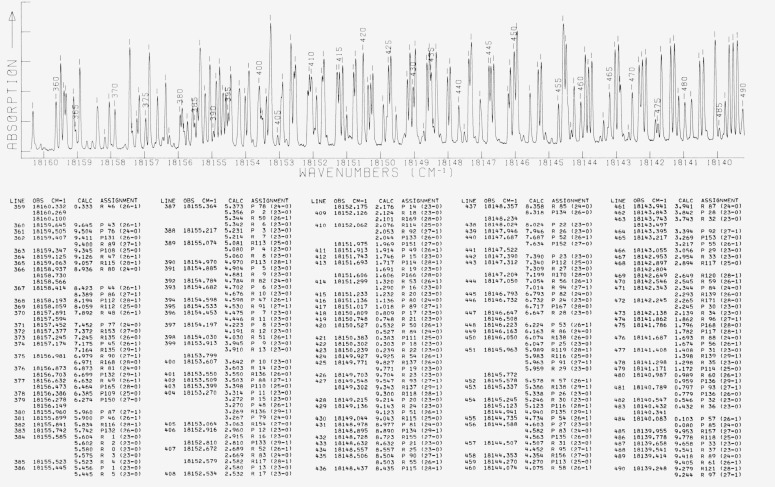

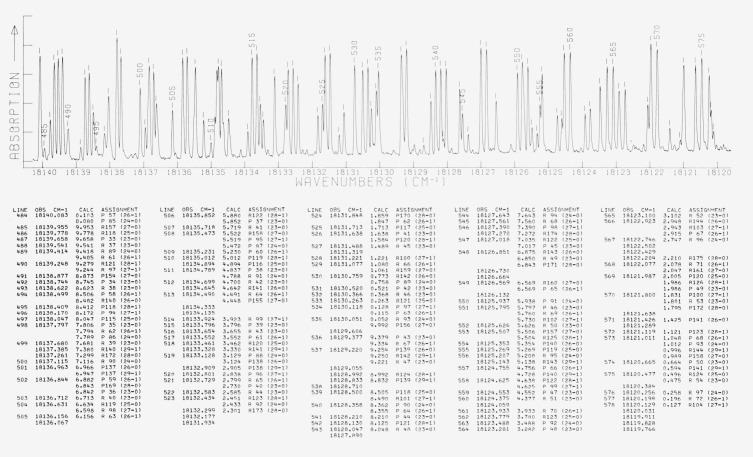

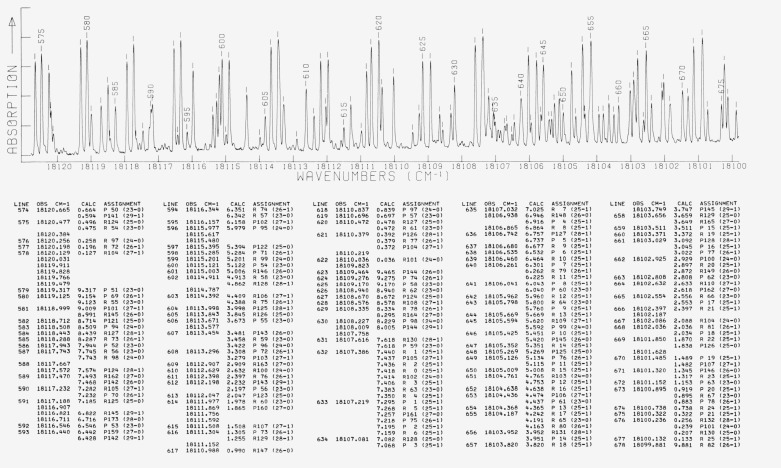

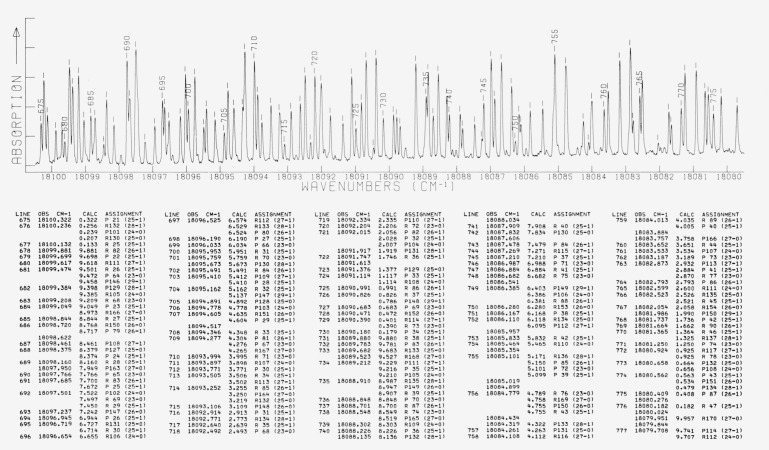

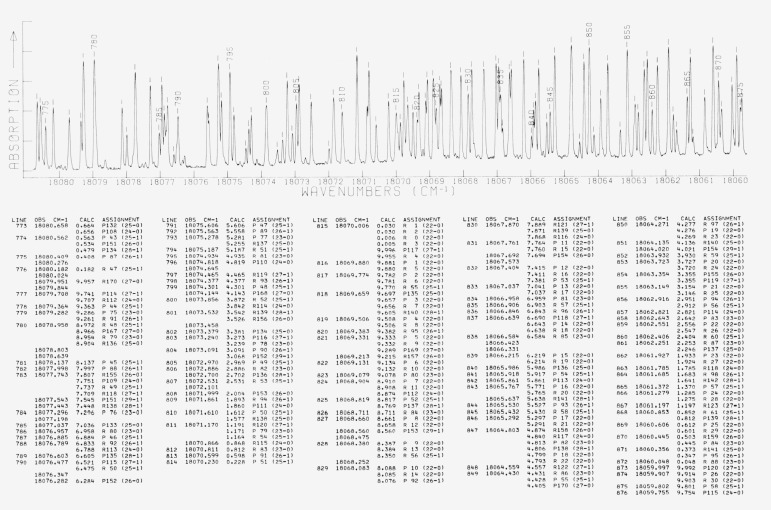

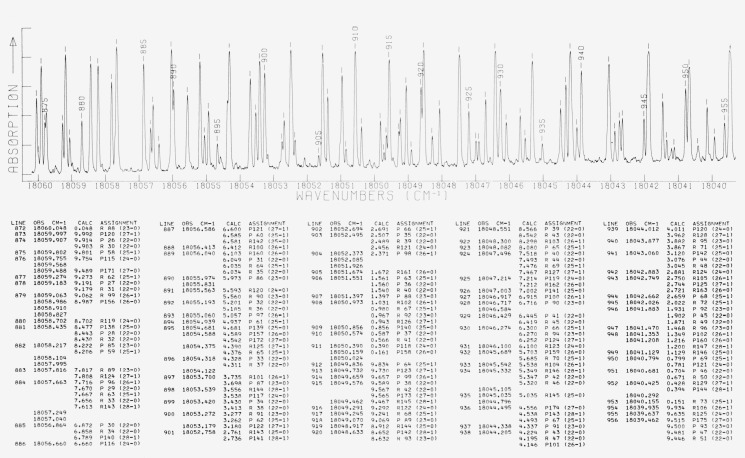

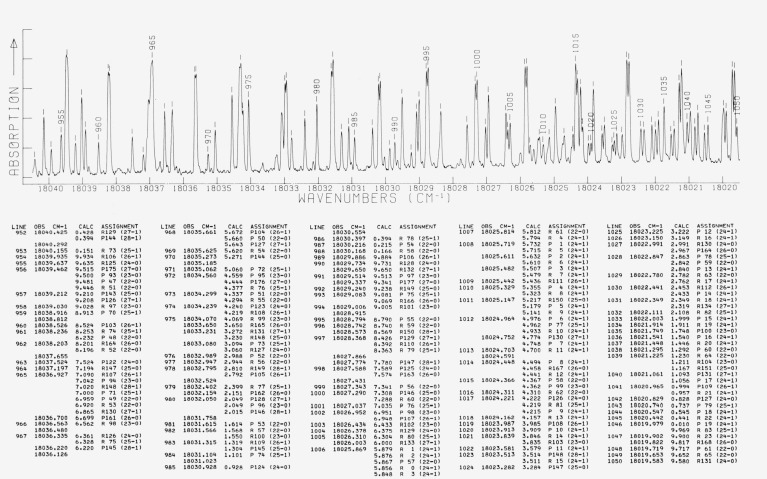

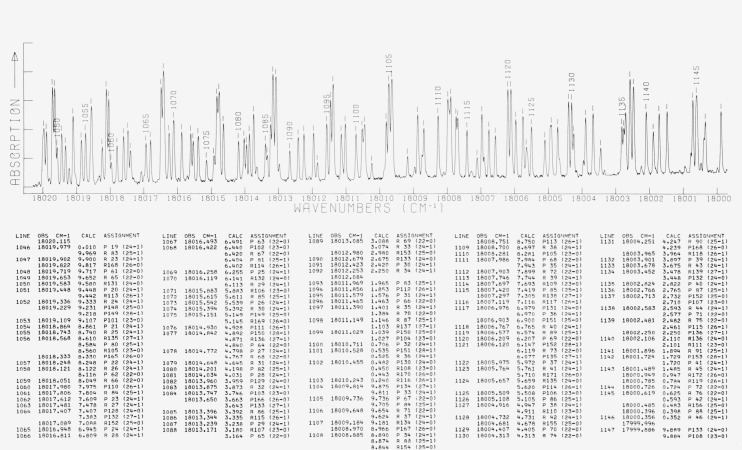
Line identification atlas of the *I_2_* absorption spectrum from 19 000 to 18 000 cm*^−1^*. See section 3 of the text for details.

The actual band atlas, presented below in figure 2, consists of 50 pages, each containing a 20 cm^−1^ portion of the spectrum, augmented by a 0.5 cm^−1^ overlap at each end. The figure at the top of each page is a CALCOMP display of a photodensitometer trace of the original photographic record of the spectrum. The tabular material below each spectral trace contains a line identification number, a measured wavenumber, the last four digits of a calculated wavenumber, a rotational assignment, and a vibrational assignment. Measured wavenumbers are presented in the atlas in decreasing numerical order, corresponding to the established optical spectroscopy prescription of “red to the right.” More detailed comments on the atlas are presented in section 3 below.

## 2. Apparatus^2^

Each I_2_ band for which absorption lines fall in the region of the atlas has been photographed, measured and assigned in its entirety. Therefore, the bands actually analyzed extend from about 19 500 to 17 700 cm^−1^.

The spectral plates were photographed in the 10th, 11th, or 12th order of a 3.34 m Czemy-Turner spectrograph constructed at the National Bureau of Standards by Dr. J. Reader [6, 7]. The spectrograph is equipped with a 300 line/mm, 220 mm long grating blazed at 6 *μ*m, and is capable of delivering close [8] to its theoretical resolving power (726 000 in 11th order). Unfortunately, the Doppler width of I_2_ (0.014 cm^−1^ FWHM at 18 500 cm^−1^ and 25 °C), the quadrupole hyperfine pattern width (~0.030 cm^−1^), and the instrumental resolution of the photodensitometer prevent this large resolving power from being fully utilized. The measured I_2_ linewidths (FWHM) in the spectra presented are of the order of 0.055 cm^−1^, corresponding to an effective resolving power of approximately 350 000. Exposure times with a high-pressure xenon source lamp and Kodak V-F plates varied from 5 to 20 min. Iodine pressure in the room-temperature, single-pass, 1-m absorption cell was controlled by a sidearm cooled to temperatures in the range −11 °C to + 6 °C. Iodine sidearm temperatures and exposure times are indicated in table 1 for each of the five plates used in the spectral illustrations below.

The I_2_ spectrum was measured against thorium emission line standards taken from the extensive catalog of R. Zalubus [9, 10]. Many of the stronger lines have been interferometrically measured, and thorium exposure times were kept short enough to eliminate most of the weaker “grating” lines, but long enough to insure from 20 to 40 standards across a plate encompassing 250 cm^−1^. Unfortunately, exposure times could not be reduced enough to eliminate all problems with self-reversal, which the computer software described below was not equipped to handle. The interferometrically measured thorium lines are thought to be reliable to ±0.002 cm^−1^ [10], but our third order polynomial fits across one plate (as well as the somewhat higher order fits also examined) gave standard deviations near 0.0045 cm^−1^. We believe our large standard deviation arises because a few of the thorium emission linewidths (FWHM) approached 0.2 cm^−1^, or about four times the I_2_ absorption linewidths. Our inability to achieve better polynomial fits to the thorium standards represents the principal limitation to obtaining more accurate measurements of the present I_2_ spectrum. Based on these considerations, we estimated the I_2_ measured wavenumbers to have an absolute accuracy of ±0.015 cm^−1^. This estimate was confirmed just prior to publication by the more accurate measurements of Gerstenkorn, Luc, and Perrin [4, 5], as shown in figure 1.

The photographic plates were measured on a Grant comparator, which automatically digitally recorded on magnetic tape photodensitometer readings at equidistant 3 *μ*m intervals (about 1/20 of the I_2_ FWHM) for both the unknown (I_2_) and standard (thorium) channels. The photodensitometer slit width was equivalent to approximately 9 *μ*m on the photographic plates. The magnetic tape record of the photographic plate density was then reduced to a sequentially numbered I_2_ line list in cm^−1^ and a CALCOMP spectral trace, using slightly modified versions of computer programs originally written by Dr. A. Maki [11] for reducing infrared data. Subroutines in his programs automatically locate the centers of absorption or emission lines in the two channels, fit the unknown channel against the standard channel, and invoke various criteria (excessive breadth, weakness, etc.) to eliminate undesirable lines from further consideration.

## 3. Detailed Remarks on the Atlas

To the extent practical, spectra are reproduced in figure 2 with a wavenumber scale equal to 1 cm^−1^ per cm.

The intensity scale is rather arbitrary. Iodine pressures and exposure times were chosen to minimize saturation of the strongest lines and maximize contrast between bands originating in the *v*″ = 0 level and bands originating in *v″* = 1 and 2. Nonetheless, an intensity alternation approximating the theoretical value of 7:5 for odd:even values of *J* is clearly visible in unblended portions of both the strong and weak branches.

Unfortunately, it proved impossible to photograph and develop an entire set of plates without encountering some small pinholes and/or scratches in the emulsion, which ultimately show up as apparent absorption lines in the CALCOMP spectrum. It was decided to present as large a portion of spectrum as possible from a single plate in order to preserve as much relative intensity information as possible, rather than to present only blemish-free regions from a large number of plates. We have thus attempted to locate as many of these false absorption lines as possible, by examining each plate for blemishes and by comparing CALCOMP spectra obtained from different plates. We have indicated the “correct” spectrum in the region of false absorption lines thus identified by hand-drawn dotted lines.

The columns headed LINE contain the artitrary sequential line identification number for “ticked” lines in the spectral figures, or contain a blank for “unticked” lines.

The columns headed OBS CM-1 contain measured wavenumbers for each line. Measurements for ticked lines were obtained by processing the spectrum actually shown in the figure. Measurements for unticked lines were obtained from other plates, taken at significantly higher iodine pressures to enhance the weaker lines without concern for the attendant saturation and broadening of the stronger lines. Occasional asterisk entries in this column indicate a line clearly visible in the spectral figure for which an assignment and calculated value, but no measurement, is available.

Each column headed CALC contains the last four digits of calculated wavenumbers approximately equal to the measured wavenumbers in the OBS column immediately to the left. If more than one transition is calculated to lie within the contour of a given measured line in the spectral figure, these several calculated values are given in order of decreasing wavenumber immediately to the right and below the measured line in question. For each branch of the (*v′ –* 0) and (*v′ –* 1) bands, no calculated transitions are presented having *J* values above the last observed line in the branch (as discussed below). For each branch of the (*v′ –* 2) bands and the (31 – 1) band, no calculated transitions are presented having *J* values below the first observed line or above the last observed line in the branch. Occasional asterisk entries in this column indicate false absorption lines introduced by the emulsion blemishes described above.

The columns headed ASSIGNMENT contain the rotational branch (P,R) and *J* assignment followed by the vibrational (*v*′ *– v″*) assignment of the calculated wavenumber immediately to the left, or contain the word ARTIFACT to indicate a false absorption line.

In almost all cases, the contour of a given spectral line can be understood by taking into account the one or more calculated values associated with it, together with intensity information obtained from examination of nearby unblended lines in the same branch(es).

Calculated wavenumbers presented in the CALC columns were obtained from least squares fits of unblended *P* and *R* branch lines for each individual (*v′ – v″*) band. Unblended lines were chosen by visual inspection, taking into consideration the intensity alternation and overall intensity variation expected within a given branch, and the essentially constant linewidth expected in each spectral region. For each branch of each band it proved impossible to find unblended lines below a certain minimum *J* value, and impossible to find lines at all above a certain maximum *J* value. Thus, for any branch, three types of calculated values can be defined: those interpolated between the minimum and maximum *J* values used in the fit, those extrapolated to low *J* beyond lines used in the fit, and those extrapolated to high *J* beyond lines used in the fit. In no cases are calculated values corresponding to high *J* extrapolations presented in this atlas. For (*v*′ – 0) and most (*v*′ *–* 1) bands, all calculated values corresponding to low *J* extrapolations are presented. For the much weaker (*v*′ *^–^* 2) bands and the (31 – 1) band no calculated values corresponding to low *J* extrapolations are presented.

Least squares fits of the unblended lines in each individual (*v*′ – *v″*) band were carried out by varying the parameters *v*_0_, *B*′, *B″*, *D*′, *D″, H*′, *H″* and sometimes *L*′ in equations of the form

$$\begin{array}{l} R\left(J\right)=+B^{\prime }{\;}_{v^{\prime }}\left(J+1\right)\left(J+2\right)-D^{\prime }{\;}_{v^{\prime }}{\left(J+1\right)}^{2}{\left(J+2\right)}^{2}\\ \;+H^{\prime }{\;}_{v^{\prime }}{\left(J+1\right)}^{3}{\left(J+2\right)}^{3}-L^{\prime }{\;}_{v^{\prime }}{\left(J+1\right)}^{4}{\left(J+2\right)}^{4}\\ \;-B^{\prime \prime }{\;}_{v^{\prime \prime }}J\left(J+1\right)+D^{\prime \prime }{\;}_{v^{\prime \prime }}J^{2}{\left(J+1\right)}^{2}\\ \;-H^{\prime \prime }{\;}_{v^{\prime \prime }}J^{3}{\left(J+1\right)}^{3}+v_{0}\left(v^{\prime },v^{\prime \prime }\right)\\ \;P\left(J\right)=+B^{\prime }{\;}_{v^{\prime }}J\left(J-1\right)-D^{\prime }{\;}_{v^{\prime }}J^{2}{\left(J-1\right)}^{2}\\ \;+H^{\prime }{\;}_{v^{\prime }}J^{3}{\left(J-1\right)}^{3}+L^{\prime }{\;}_{v^{\prime }}J^{4}{\left(J-1\right)}^{4}\\ \;-B^{\prime \prime }{\;}_{v^{\prime \prime }}J\left(J+1\right)+D^{\prime \prime }{\;}_{v^{\prime \prime }}J^{2}{\left(J+1\right)}^{2}\\ \;-H^{\prime \prime }{\;}_{v^{\prime \prime }}J^{3}{\left(J+1\right)}^{3}+v_{0}\left(v^{\prime },v^{\prime \prime }\right). \end{array}$$ (1)

Values of the parameters and standard deviations obtained from these band-by-band least squares fits, and of *J*_min_ *J*_max_ and the number of lines in each branch included in the fit, are given in table 2. *The reader is emphatically warned that these band-by-band parameters must not be treated as true molecular constants*. In particular, they should not be used to extrapolate branches beyond *J* values used in the fits (though with some misgivings we ourselves have violated this precept in presenting calculated values for all low *J* lines in the (*v*′ *–* 0) and (*v*′ *–* 1) heads). Neither should the band-by-band parameters be further reduced to obtain structural information for the I_2_ molecule. These parameters are useful, however, and have been used in this atlas, to calculate *interpolated* line positions within a branch; they are presented in table 2 with sufficient precision to permit such back-calculation to within 0.001 cm^−1^, even though this requires in all cases many more significant figures than are physically meaningful.

As a consistency check on the rotational assignments in this atlas, which as mentioned above were determined essentially by extending the calculated branches of Wei and Tellinghuisen to higher *J*, we present in table 3 a set of ground state combination differences. These Δ_2_F″(*J*) values were calculated using *v*″ = 0 parameters taken from the band-by-band least squares fits. Since measured I_2_ linewidths (FWHM) on the spectral figures are of the order of 0.055 cm^−1^, we see that calculated interpolated combination differences agree to 1/20 of the FWHM for *J* < 150 and to 1/5 of the FWHM for higher *J*.

As a further consistency check, Dr. M. M. Hessel [12] has kindly least squares fit 5741 unblended lines assigned in this work to a 29-parameter Dunham expansion, obtaining an overall standard deviation of 0.0042 cm^−1^. Such a fit introduces only one set of rotational constants for each vibrational level, and furthermore requires these rotational constants to vary smoothly with vibrational quantum number. The Dunham coefficients obtained are close to true molecular constants, but are not given here since the “best” values for such constants must be determined from a fit of the unblended lines from the entire visible spectrum of I_2_, rather than from a 1000 cm^−1^ portion.

Unfortunately, no independent support for the vibrational assignments arose from the work for this atlas.

## Figures and Tables

**Table 1:** Iodine sidearm temperatures and exposure times for the spectral figures in this atlas

Spectral region	*T* °C	Exposure time
19 000–18 920	−6	5 minutes
18 920–18 640	−6	7 minutes
18 640–18 380	−6	5 minutes
18 380–18 180	−11	6.5 minutes
18 180–18 000	−6	6.5 minutes

**Table 2:** Interpolation parameters^~^ obtained from least squares fits of individual (v′-v″) bands to equations (1).

v′-v″	P Branch			R Branch											
	J_min_	J_max_	No. Lines Fit	J_min_	J_max_	No. Lines Fit	Std. Dev. of Fit	v_0_ × 10^−5^	B′ × 10^1^	B″ × 10^1^	D′ × 10^7^	D″ × 10^8^	H′ × 10^13^	H″ × 10^14^	L′ × 10^18^
							(cm^−1^)	(cm^−1^)	(cm^−1^)	(cm^−1^)	(cm^−1^)	(cm^−1^)	(cm^−1^)	(cm^−1^)	(cm^−1^)
45-0	11	167	73	13	163	77	0.0024	0.195163613	0.18018728	0.37299442	0.277981	0.33354	−1.34323	−3.1957	−1.8958
44-0	22	157	75	7	161	72	0.0022	0.194759905	0.18394367	0.37313127	0.279128	0.46582	−0.88318	0.1893	−1.6130
43-0^b^	27	152	52	10	163	63	0.0027	0.194336823	0.18722787	0.37284063	0.246293	0.22560	−1.12772	−5.4578	−1.8808
42-0	12	175	87	14	174	82	0.0024	0.193894140	0.19094128	0.37308710	0.248824	0.43113	−0.95171	−0.5887	−0.9056
41-0	7	171	81	9	173	94	0.0025	0.193431465	0.19436945	0.37307194	0.233181	0.39122	−1.03320	−1.7714	−0.6655
40-0	8	171	94	6	173	92	0.0022	0.192948559	0.19786561	0.37315117	0.234316	0.46486	−0.57164	−0.1072	−0.9103
39-0	5	171	95	5	169	88	0.0021	0.192445316	0.20116427	0.37311621	0.224764	0.45068	−0.38998	−0.3671	−1.0901
38-0	7	175	84	7	178	90	0.0020	0.191921469	0.20453472	0.37322028	0.224938	0.54155	−0.01774	1.9019	−1.2150
37-0	5	178	90	6	177	100	0.0022	0.191376895	0.20762792	0.37313737	0.201702	0.45137	−0.43993	−0.4603	−0.7966
36-0	4	170	92	5	178	111	0.0023	0.190811385	0.21082957	0.37320255	0.198457	0.54288	−0.37426	1.9948	−0.3438
35-0	5	179	105	5	169	107	0.0021	0.190224898	0.21385144	0.37312974	0.183564	0.48768	−0.56247	0.9779	
34-0	9	176	98	12	179	99	0.0019	0.189617408	0.21683688	0.37306535	0.170785	0.41564	−0.64132	−0.8193	
33-0	13	174	85	14	181	107	0.0025	0.188988936	0.21976994	0.37303719	0.161443	0.39176	−0.65357	−1.5785	
32-0^C^	7	172	71	6	179	88	0.0023	0.188339425	0.22270804	0.37309719	0.156107	0.42573	−0.58491	−0.9737	
31-0^d^	31	172	70	5	178	74	0.0028	0.187668755	0.22521063	0.37275425	0.132334	0.25073	−0.80653	−3.6445	
30-0	11	179	97	7	175	90	0.0015	0.186977046	0.22836349	0.37311068	0.147624	0.45179	−0.39097	−0.1603	
29-0	7	170	101	5	179	112	0.0016	0.186264451	0.23105991	0.37308489	0.138863	0.41706	−0.42877	−1.0541	
28-0	6	173	95	4	175	94	0.0017	0.185530998	0.23372552	0.37309320	0.133996	0.42405	−0.39673	−1.0795	
27-0	5	177	107	5	170	114	0.0024	0.184776805	0.23640338	0.37318390	0.136129	0.51231	−0.17679	1.1841	
26-0	5	171	114	10	172	110	0.0017	0.184001954	0.23892968	0.37316711	0.129675	0.50398	−0.18282	1.1061	
25-0	7	177	119	7	179	111	0.0015	0.183206599	0.24133627	0.37305832	0.119061	0.42247	−0.28070	−0.5431	
24-0	8	168	116	8	174	112	0.0015	0.182391040	0.24375738	0.37304948	0.113625	0.40877	−0.28981	−0.9579	
23-0	17	169	94	5	172	92	0.0017	0.181555307	0.24621734	0.37313994	0.115061	0.46874	−0.16403	0.1433	
22-0^e^	15	164	65	5	170	71	0.0027	0.180699558	0.24864723	0.37324815	0.117127	0.53405	−0.05612	1.2097	
31-1	34	123	17	27	137	20	0.0021	0.185535703	0.22570051	0.37207962	0.159268	0.50413	−0.22472	1.8374	
30-1	9	146	38	12	129	33	0.0020	0.184844042	0.22840061	0.37199561	0.149532	0.45175	−0.34218	−0.6779	
29-1	8	154	38	7	145	44	0.0026	0.184131473	0.23120341	0.37210813	0.152554	0.58087	−0.05690	3.1710	
28-1	12	154	52	8	150	34	0.0023	0.183397930	0.23380043	0.37202312	0.145406	0.55583	−0.01508	3.3062	
27-1	7	163	52	7	156	52	0.0016	0.182643739	0.23631178	0.37191287	0.131330	0.44247	−0.25016	−0.0205	
26-1	6	156	43	7	157	43	0.0012	0.181869034	0.23888172	0.37196383	0.126468	0.45627	−0.25907	−0.1474	
25-1	5	157	57	8	157	69	0.0015	0.181073699	0.24139129	0.37198067	0.123166	0.46501	−0.21543	−0.1149	
24-1	10	167	71	5	161	76	0.0017	0.180258069	0.24382636	0.37198675	0.120973	0.49687	−0.10304	1.1659	
37-2	11	114	21	12	114	25	0.0023	0.187123221	0.20767058	0.37091688	0.216472	0.68350	−0.00006	9.1877	
36-2	8	110	25	11	105	20	0.0026	0.186557601	0.21072968	0.37073115	0.193327	0.37002			
35-2	11	107	21	10	106	20	0.0024	0.185971212	0.21404409	0.37097251	0.202416	0.55783			
34-2	15	109	18	33	108	16	0.0026	0.185363708	0.21686690	0.37075985	0.181556	0.42558			
33-2	24	115	20	40	109	21	0.0024	0.184735201	0.22001681	0.37096045	0.191667	0.62031			

a The parameters in this table are to be used only for calculating interpolated line positions in the P and R branches of individual (v′-v″) bands, and are presented here with sufficient significant figures to permit this back calculation to within 0.001 cm^−1^ These parameters are not to be interpreted as molecular constants, and are thus not given with standard deviations, which in all cases correspond to errors considerably greater than implied by the number of significant figures presented in this table.

b P and R branches blended from the band origin to P(60) and R(62).

c P and R branches blended from the band origin to P(35) and R(38).

d P and R branches blended from the band origin to P(92) and R(95).

e P and R branches blended from the band origin to P(59) and R (63).

**Table 3:** Lower state combination differences, Δ_2_F″(J), for the (v′-0) bands calculated from the constants of Table 2 for J values below that of the last transition used in the least squares fit.

J	(45-0)	(44-0)	(43-0)^a^	(42-0)	(41-0)	(40-0)	(39-0)	(38-0)	(37-0)	(36-0)	(35-0)	(34-0)
10	1.567	1.567	1.566	1.567	1.567	1.567	1.567	1.568	1.567	1.567	1.567	1.567
20	3.058	3.059	3.057	3.059	3.059	3.060	3.059	3.060	3.059	3.060	3.059	3.059
30	4.550	4.551	4.548	4.551	4.551	4.551	4.551	4.552	4.551	4.552	4.551	4.551
40	6.041	6.042	6.039	6.042	6.042	6.043	6.042	6.043	6.042	6.043	6.042	6.041
50	7.531	7.533	7.529	7.532	7.532	7.533	7.532	7.534	7.533	7.533	7.532	7.532
60	9.020	9.022	9.018	9.021	9.021	9.022	9.021	9.023	9.022	9.022	9.021	9.021
70	10.508	10.509	10.507	10.509	10.509	10.510	10.509	10.510	10.510	10.510	10.509	10.509
80	11.995	11.996	11.994	11.995	11.996	11.996	11.995	11.996	11.996	11.995	11.995	11.995
90	13.480	13.480	13.480	13.480	13.481	13.480	13.480	13.480	13.481	13.479	13.479	13.480
100	14.963	14.962	14.963	14.962	14.964	14.963	14.962	14.962	14.963	14.961	14.961	14.963
110	16.444	16.443	16.444	16.443	16.444	16.443	16.442	16.442	16.443	16.441	16.442	16.443
120	17.922	17.920	17.923	17.921	17.922	17.921	17.920	17.919	17.921	17.919	17.920	17.921
130	19.397	19.396	19.397	19.396	19.397	19.395	19.395	19.394	19.395	19.394	19.395	19.396
140	20.867	20.868	20.868	20.868	20.868	20.867	20.867	20.867	20.867	20.867	20.868	20.869
150	22.334	22.337	22.333	22.337	22.336	22.336	22.335	22.338	22.336	22.337	22.339	22.338
160	23.795			23.802	23.799	23.801	23.800	23.806	23.800	23.806	23.806	23.803
170				25.263	25.258	25.263	25.262	25.272	25.261	25.272	25.271	25.264

J	(33-0)	(32-0)^b^	(31-0)^c^	(30-0)	(29-0)	(28-0)	(27-0)	(26-0)	(25-0)	(24-0)	(23-0)	(22-0)^d^
10	1.567	1.567	1.566	1.567	1.567	1.567	1.567	1.567	1.567	1.567	1.567	1.568
20	3.059	3.059	3.056	3.059	3.059	3.059	3.060	3.060	3.059	3.059	3.059	3.060
30	4.550	4.551	4.547	4.551	4.551	4.551	4.552	4.552	4.550	4.550	4.551	4.552
40	6.041	6.042	6.037	6.042	6.042	6.042	6.043	6.043	6.041	6.041	6.042	6.044
50	7.531	7.532	7.527	7.532	7.532	7.532	7.533	7.533	7.531	7.531	7.533	7.534
60	9.020	9.021	9.016	9.021	9.021	9.021	9.022	9.022	9.021	9.021	9.022	9.023
70	10.508	10.509	10.504	10.509	10.509	10.509	10.510	10.509	10.508	10.508	10.509	10.511
80	11.995	11.996	11.991	11.995	11.996	11.996	11.996	11.995	11.995	11.995	11.996	11.997
90	13.480	13.480	13.476	13.480	13.480	13.480	13.480	13.480	13.479	13.480	13.480	13.481
100	14.962	14.963	14.960	14.962	14.963	14.963	14.962	14.962	14.962	14.962	14.962	14.963
110	16.443	16.443	16.442	16.442	16.443	16.443	16.442	16.442	16.443	16.443	16.443	16.442
120	17.921	17.921	17.921	17.920	17.921	17.920	17.919	17.920	17.921	17.921	17.920	17.920
130	19.396	19.396	19.397	19.395	19.396	19.395	19.395	19.395	19.396	19.396	19.395	19.394
140	20.867	20.867	20.869	20.868	20.868	20.867	20.867	20.867	20.869	20.868	20.867	20.866
150	22.335	22.335	22.338	22.337	22.336	22.335	22.337	22.338	22.338	22.337	22.337	22.335
160	23.799	23.800	23.801	23.802	23.801	23.799	23.804	23.805	23.804	23.802	23.802	23.801
170	25.259	25.260	25.259	25.264		25.258	25.269	25.269	25.266			

a P and R branches blended from the band origin to P(60) and R(62).

b P and R branches blended from the band origin to P(35) and R(38).

c P and R branches blended from the band origin to P(92) and R(95).

d P and R branches blended from the band origin to P(59) and R(63).

## Footnotes

1 Figures in brackets indicate the literature references at the end of this paper.

2 In order to adequately describe materials and experimental procedures, it was occasionally necessary to identify commercial products by manufacturer’s name or label. In no instance does such identification imply endorsement by the National Bureau of Standards, nor does it imply that the particular product or equipment is the best available for the purpose.
